# Gender Differences in the Acute Kidney Injury to Chronic Kidney Disease Transition

**DOI:** 10.1038/s41598-017-09630-2

**Published:** 2017-09-25

**Authors:** Ixchel Lima-Posada, Cinthya Portas-Cortés, Rosalba Pérez-Villalva, Francesco Fontana, Roxana Rodríguez-Romo, Rodrigo Prieto, Andrea Sánchez-Navarro, Guadalupe L. Rodríguez-González, Gerardo Gamba, Elena Zambrano, Norma A. Bobadilla

**Affiliations:** 1 Molecular Physiology Unit, Instituto de Investigaciones Biomédicas, Universidad Nacional Autónoma de México, Mexico City, Mexico; 2 Departament of Nephrology, Instituto Nacional de Ciencias Médicas y Nutrición Salvador Zubirán, Mexico City, Mexico; 3 Departament of Reproductive Biology, Instituto Nacional de Ciencias Médicas y Nutrición Salvador Zubirán, Mexico City, Mexico

## Abstract

This study evaluated if there is a sexual dimorphism in the acute kidney injury (AKI) to chronic kidney disease (CKD) transition and the time-course of the potential mechanisms involved in the dimorphic response. Female and male rats were divided into sham-operated or underwent 45-min renal ischemia (F + IR, and M + IR). All groups were studied at 24-h and 1, 2, 3, or 4-months post-ischemia. Additionally, oophorectomized rats were divided into sham or IR groups. After 24-h, AKI extent was simllar in females and males, but female rats exhibited less oxidative stress and increased renal GSH content. After 4-months and despite similar AKI, the M + IR group developed CKD characterized by proteinuria, tubulointerstitial fibrosis, glomerular hypertrophy, increased oxidative stress and a reduction in HIF1α and VEGF from the 1^st^-month and persisting throughout the time-course studied. Interestingly, the F + IR group did not develop CKD due to lesser oxidative stress and increased eNOS, TGFβ and HIF1α mRNA levels from the 1^st^-month after IR. Whereas, oophorectomized rats did develop CKD. We found a sexual dimorphic response in the AKI to CKD transition. Early antioxidant defense and higher TGFβ, HIF1α and eNOS were among the renoprotective mechanisms that the F + IR group demonstrated.

## Introduction

Renal ischemia/reperfusion injury (IRI) is a major cause of acute kidney injury (AKI) in patients hospitalized with native or transplanted kidneys^1,2^. It affects 15% of hospitalized patients, and the highest incidence is found in patients in the intensive care unit, with up to 60% of patients affected^2–4^. Ischemic AKI is provoked by a reduction in renal blood flow (RBF)^3,5^, producing endothelial and tubular epithelial injury^6–8^. Consequently, peritubular capillary perfusion is also reduced, which favors the harm of S2 and S3 segments of the proximal tubule due to the large number of mitochondria in these sections. Therefore, this segment is highly susceptible to oxygen tension changes, with the Na^+^/K^+^ ATPase as one of most affected enzymes. Likewise, a reduction in ATP produces uncoupling of the respiratory chain and the subsequent formation of free radicals that favor the detachment of epithelial cells and death by apoptosis or necrosis^9,10^.

Although the tubular epithelium can recover from lethal or sublethal cell damage, cellular processes in endothelial and tubular cells may not fully recover, thereby conditioning the development of progressive renal dysfunction^11^.

In the last two decades, the incidence of CKD has increased more than three times, and according to the World Health Organization (WHO), it will be one of the three leading causes of death and disability worldwide by 2020^12^. This will certainly impact health systems around the world. CKD is characterized by progressive loss of nephrons and renal function, in which tubule-interstitial fibrosis plays an important role^13,14^. Accumulated evidence during the last decade from epidemiological and experimental observations have revealed that AKI is an important risk factor for the development of CKD and may also promote the CKD transition to end stage renal disease (ESRD)^8,14–17^. However, few studies have addressed the mechanisms of AKI transition to CKD. Several theories have tried to explain how an episode of AKI leads to renal function and structure injury over time. These theories include repeated cycles of damage and repair^18^, rarefaction of peritubular capillaries with the subsequent development of chronic hypoxia^7,19,20^, and activation of signaling pathways such as hypoxia inducible factor (HIF1), pro-fibrotic and pro-inflammatory cytokines^21,22^. In addition, although it is essential that the tubular cells proliferate to restore normal tubular structure, studies suggest that epithelial cells of the renal tubules also play a critical role in the development of tubulointerstitial fibrosis by inducing an arrest in the cell cycle, causing disproportionate tubular proliferation, TGFβ generation, and epigenetic modifications^23–26^.

Moreover, there is a growing evidence that the pathogenesis, clinical features and prognosis of cardiovascular and renal diseases is completely different between men and women, which makes sense since the physiology of women is different from men. In this sense, one of the largest meta-analysis to assess gender differences in the progression of renal diseases included 11,000 patients referred from 60 different studies and showed that women demonstrate lower progression compared to men in different renal diseases, such as polycystic kidney disease, IgA nephropathy, membranous glomerulonephritis and CKD^27^. In support of this meta-analysis, two recent studies showed that the progression to CKD is worse in men than in women^28,29^. Likewise, the PREVEND cohort revealed that age, albuminuria, body mass index and blood glucose levels in men are risk factors that exacerbate the progression to ESRD at a greater magnitude than in women^30^.

In this study, we evaluated whether there is a sexual dimorphism in the AKI to CKD transition, the time-course of functional and structural alterations in both genders, the effect of oophorectomy on this transition, and the mechanisms responsible for gender differences.

## Results

**Figure 1:**
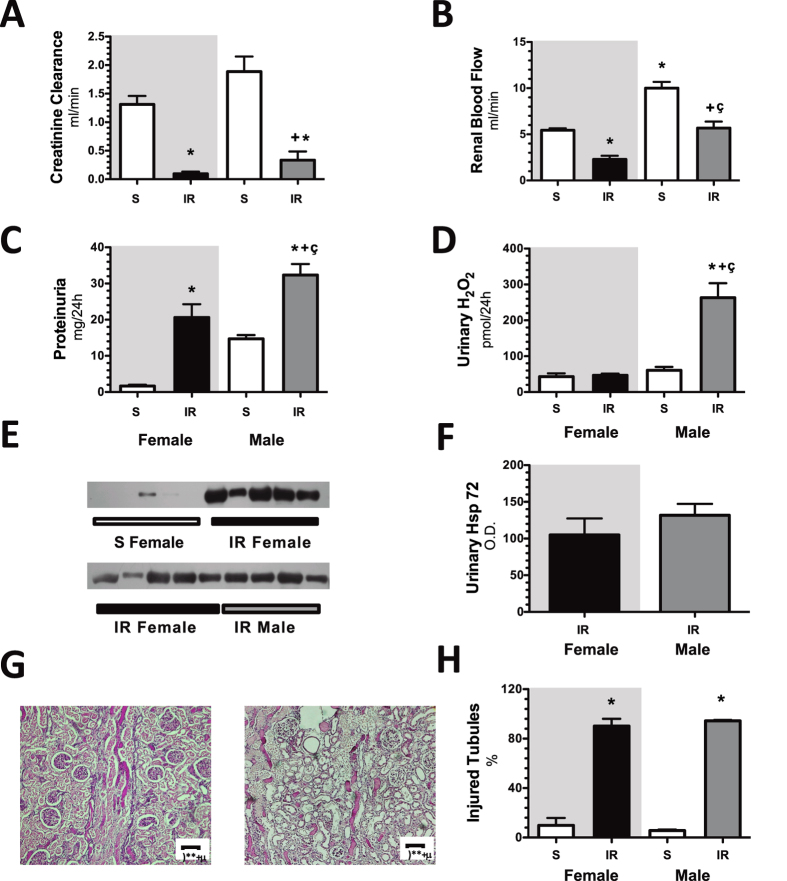
Renal injury induced by ischemia/reperfusion after 24-h in both female and male rats. (**A**) Creatinine clearance, (**B**) renal blood flow, (**C**) proteinuria, (**D**) Urinary H_2_O_2_ excretion, (**E**) Urinary Hsp72 levels by Western blot (n = 4–5 per group). (**F**) Densitometric analysis of Hsp72 levels, (**G**) a representative image of a periodic acid–Schiff (PAS) stained kidney slides from a female rat underwent IR (left), and an IR male rat (right), (**H**) percentage of injured tubules. Female groups are in a gray background in which sham female is represented by white bars and IR female group in black bars. Following by sham male in white bars and IR male group in gray bars. Control groups were formed at least n = 5, and IR Female or Male = at least 6. Data are shown as mean ± SE. *p < 0.05 vs, Sham female group, ^+^p < 0.05 vs. sham male group, and ^ç^p < 0.05 vs. F + IR group.

**Figure 2:**
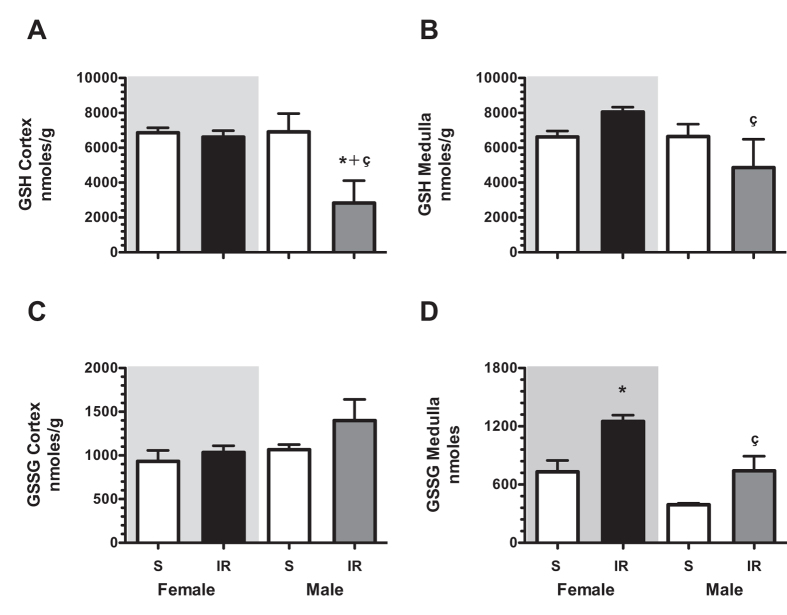
The IR renal injury induced a dimorphic GSH and GSSG kidney content response. (**A**) Cortex GSH levels, (**B**) Medulla GSH levels, (**C**) Cortex GSSG levels, (**D**) Medulla GSSG levels. Female groups are in a gray background in which Sham female is represented by white bars and IR female group in black bars. Following by sham male in white bars and IR male group in gray bars. The GSH and GSSG kidney content was evaluated 24-h post-ischemia. Control groups were formed by n = 4, whereas F + IR and M + IR groups included n = 6. Data are shown as mean ± SE. *p < 0.05 vs, Sham female group, ^+^p < 0.05 vs. sham male group, and ^ç^p < 0.05 vs. F + IR group.

Our first target was focused on assessing the severity IRI in male and female rats. The AKI was induced by 45 min of ischemia and compared with the control groups. These groups were studied 24-h after IR. The M + IR and F + IR groups developed AKI, characterized by a significant reduction in creatinine clearance (Fig. 1A), and renal blood flow (Fig. 1B), together with a significant increase in proteinuria (Fig. 1C). The urinary excretion of hydrogen peroxide (UH_2_O_2_V) increased 4-fold in the M + IR group compared to its control group, interestingly, in the F + IR group, the UH_2_O_2_V remained unaltered (Fig. 1D). Previously our group showed that the urinary heat shock protein 72 kDa levels (UHsp72) is a sensitive and early biomarker of AKI, capable of stratifying the intensity of epithelial tubular damage^31^. As shown in Fig. 1E and F, the female control group showed almost no UHsp72. In contrast, the F + IR group showed a significant increase in UHsp72 (upper WB). Similar levels of Hsp72 were found in the M + IR and F + IR groups (lower WB and densitometric analysis). This similar degree of IRI was confirmed by the histopathologic analysis as is shown in the representative PAS-stained kidney slices and in the quantification of injured tubules (Fig. 1G–H). These results indicate that IRI caused structural and functional alterations of the same magnitude in male and female rats, except in UH_2_O_2_V. The dimorphic response observed in the UH_2_O_2_V was further analyzed by measuring glutathione levels (GSH) into the kidney (Fig. 2). In renal cortex, GSH content was similar in control and the F + IR groups. In contrast, the M + IR group displayed a significant reduction by 60% in the GSH levels compared to both control groups. (Fig. 2A). In renal medulla, GSH content was similar in both control groups, whereas the F + IR group exhibited greater GSH content by t-test (p = 0.01) but it not reach statistical difference by ANOVA. In contrast, the M + IR group exhibited lesser GSH levels compared to F + IR group (Fig. 2B). The GSSG levels in the renal cortex, a subrogate metabolite of GSH oxidation, we only observed a trend to increase in the M + IR group (Fig. 2C). Whereas in the renal medulla, GSSG levels were greater in the F + IR group (Fig. 2D). These findings indicate that despite similar IRI female rats had a greater ability to generate GSH.

**Figure 3:**
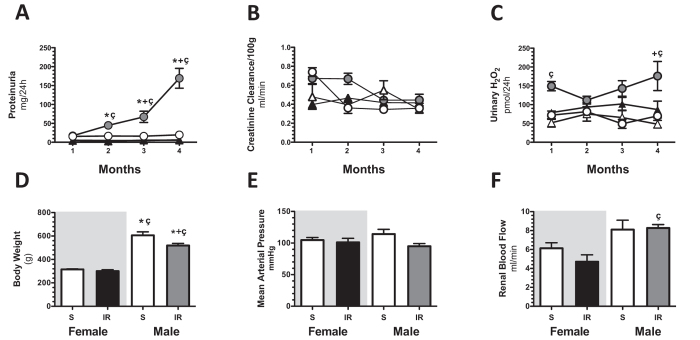
The AKI to CKD transition is prevented in female rats. Every 30-days (**A**) Urinary protein excretion, (**B**) creatinine clearance and (**C**) Urinary H_2_O_2_ excretion were measured. White triangles represent Sham female (n = at least 7), black triangles represent IR female (n = at least 10), white circles represent Sham male (n = at least 4) and gray circles represent IR male group (n = at least 4). After 4 months, (**D**) body weight, (**E**) mean arterial pressure, and (**F**) renal blood flow were recorded. Sham Female or Male (n = at least 4) and IR Female or Male groups (n = at least 7). Female groups are in a gray background in which sham is represented by white bars and F + IR group in black bars, following by Sham male in white bars and M + IR group in gray bars. Data are shown as mean ± SE. *p < 0.05 vs, Sham female group, ^+^p < 0.05 vs. Sham male group, and ^ç^p < 0.05 vs. F + IR group.

Our second target was focused on assessing the time-course of AKI to CKD transition in female and male rats. As we previously reported^23,26,32^, an ischemic episode in male rats induced a progressive increase in proteinuria from 17.2 ± 1.4 (1^st^-month) to 169.2 ± 26.2 mg/dL (4^th^-month) and it was evident since the 2^nd^-month (Fig. 3A). The temporal course of renal function corrected by body weight was similar among the groups and did not alter by IRI (Fig. 3B). In contrast, an early increase in UH_2_O_2_V was observed in the M + IR group (1^st^-month) that remained elevated during the time-course of the study when it was compared to its control group by t-test (Fig. 3C), but only significant by ANOVA at the 1^st^ and 4^th^-month compared with F + IR group. Despite the same initial IRI, the F + IR group did not develop neither proteinuria nor elevation of UH_2_O_2_V (Fig. 3A,C).

At the end of the experimental period (4-months), both female groups exhibited a lesser body weight than male, due to the well-known biological sex difference in size and body weight. In the M + IR group there was a slight reduction that was associated with the AKI to CKD transition that exhibited the male rats, but not the females (Fig. 3D). No differences in the mean arterial pressure among the studied groups were observed (Fig. 3E). The M + IR and F + IR groups did not exhibit changes in RBF, although it was significantly higher in male than in female groups (Fig. 3F), this difference was not observed when RBF was corrected by the body weight (data not shown).

**Figure 4:**
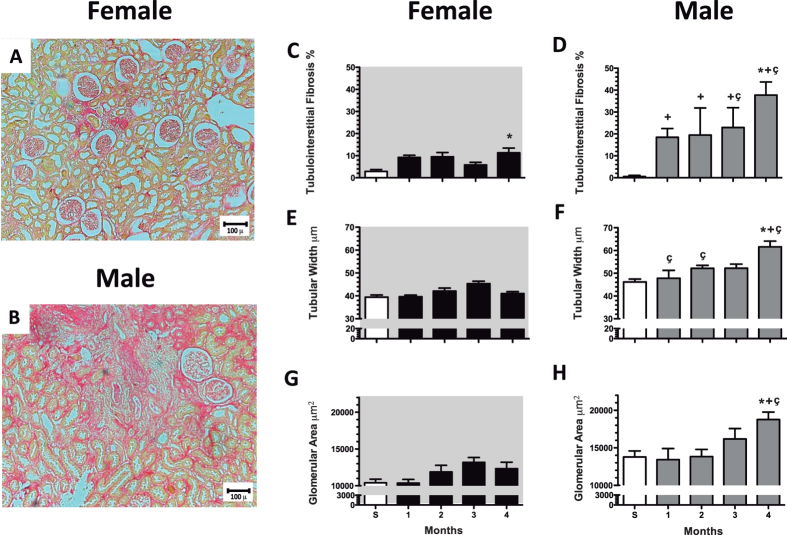
CKD induced by an AKI episode was associated with glomerular and tubulointerstitial injury in males but prevented in females. Representative light microphotographs of kidney slides stained with Sirius red from (**A**) female IR rat an (**B**) male IR rat after 4-months of IR injury (Magnification ×100). Temporal course of tubulointerstitial fibrosis in (**C**) female (white bar represents sham and black bars IR groups) and in (**D**) male groups (white bar represents sham and gray bars IR groups). Temporal course of tubular dilation (μm) in (**E**) female and in (**F**) male groups. Temporal course of Glomerular area (μm^2^) in (**G**) female and in (**H**) male groups. All parameters were determined at 1, 2, 3 and 4-months in both F + IR and M + IR groups and at 4-months in female and male sham groups in at least 4 rats per group. Data are shown as mean ± SE. *p < 0.05 vs, Sham female group, ^+^p < 0.05 vs. sham male group, and ^ç^p < 0.05 vs. F + IR group.

In accord with our findings at functional level, after the 4^th^-month, the F + IR group showed almost no renal structural alterations compared to the M + IR group. The representative microphotomicrograph from a F + IR rat (Fig. 4A) contrast with a M + IR rat microphotomicrograph (Fig. 4B), wherein the presence of a large percentage of tubulointerstitial fibrosis-affected area is clearly visible. The morphometric analysis of the time-course of tubulointerstitial fibrosis showed that 4-months post-ischemia, the F + IR (Fig. 4C) and the M + IR groups (Fig. 4D) showed a significant increase in fibrosis, however the degree of damage was much greater in males than in females, 38% vs. 12%, respectively (p < 0.05). Tubular dilataion and glomerular hypertrophy was not observed in the F + IR (Fig. 4E and G, respectively). Alterations that were do present in the M + IR group (Fig. 4F and H). Supplemental Fig. 1 shows the time-course of glomerular diameter distribution in male control group (top panel) compared with the M + IR (middle panel) and F + IR groups (lower panel). After one month, the control group exhibited a normal distribution, in which about 50% of glomeruli was found in the size range of 101–125 μm (Supplemental Fig. 1A). After 2, 3 or 4-months, in the control male group, the glomerular size increased that was in accord with the rats grow-up, so the largest percentage of glomeruli diameter was found between 126 to 150 μm and the distribution looks like a bell-shaped (Supplemental Figure 1B, C and D, respectively). In contrast, in the M + IR group there was a significant increase in the proportion of glomeruli in higher ranks to 176 μ (12%, Supplemental Figure 1H). This is in clear contrast to rats belonging to the control group, which had less than 2% in this range and none glomerulus in the range between 201–225 μm. The F + IR group had a distribution pattern like the control group (Supplemental Figures 1I–1L).

The results presented in Figs 3 and 4 and Supplemental Fig. 1 clearly show that the M + IR group developed progressive CKD, this complication was not observed in the F + IR group, despite the same AKI degree induced at the beginning of the study.

**Figure 5:**
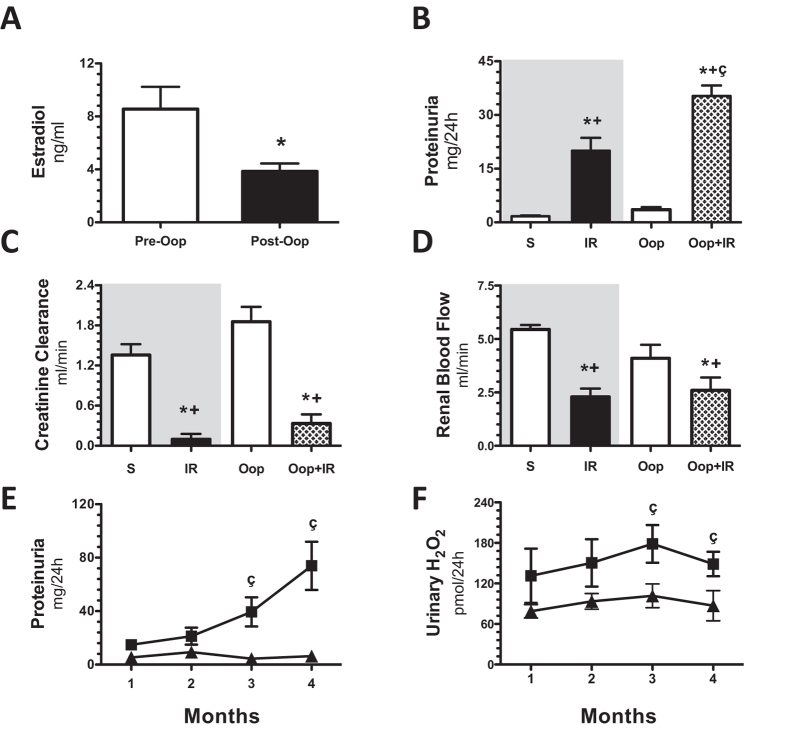
The depletion of estrogens is associated with AKI to CKD transition in female rats. (**A**) Estradiol levels before oophorectomy (Pre-Op) is represented in white bars (n = 24) and 1-month after oophorectomy (Post-Op) in black bars (n = 24). Then, oophrorectomized rats (Oop) were underwent to sham surgery or bilateral renal ischemia of 45 min (Oop + IR) and studied 24-h or 1 to 4-months. In (**B**) proteinuria, (**C**) creatinine clearance, and (**D**) renal blood flow evaluated 24-h post-ischemia. Both female groups without oophorectomy are in a gray background in which sham (S) is represented by white bars and IR female group (IR) in black bars, following by the sham oophorectomized group (Op) in white bars and the ophrorectomized underwent IR group (Oop + IR) in pattern bars. For the long-time experiment, (**E**) urinary protein excretion, and (**F**) Urinary H_2_O_2_ excretion were measured every 30 days during the follow-up. Black triangles represent IR female (n = at least 8), black squares represent IR + Oop (n = at least 4). Data are shown as mean ± SE. *p < 0.05 vs. sham female group, ^+^ p < 0.05 vs. Op, ^ç^p < 0.05 vs. F + IR group.

To dissect the renoprotective role of female sexual hormones in the long-term renoprotection observed, female rats were oophorectomized. Figure 5A shows that in oophorectomized rats one month after, estradiol levels were reduced by 55% compared with the control group, and this group of rats won more body weight than the female control rats. Although the creatinine clearance was greater in Oop group than S group the difference by ANOVA was not significant (Fig. 5C) and when the creatinine clearance was corrected by body weight, similar results were observed (0.53 ± 0.07 vs. 0.66 ± 0.08 ml/min/100 g BW, p = NS). One month after, one half of these rats underwent IR and the other half, sham surgery. After 24-h, proteinuria was much greater in Oop + IR than in the F + IR group, (Fig. 5B). The IRI was also evidenced by the reduction in creatinine clearance and renal blood flow in both groups (Fig. 5C and D, respectively). Then, the time-course of AKI to CKD was analyzed. Urinary protein excretion progressively increased in the Oop + IR group since the 3^rd^-month post-ischemia (Fig. 5E), being a pretty like the M + IR group comportment. Similarly, oxidative stress in the Oop + IR group was higher than F + IR group since the 2^nd^- month (p = 0.05), reaching a statistically significant difference in the 3^rd^ and 4^th^-months (Fig. 5F). These results suggest that female sexual hormones play a crucial role in avoiding the AKI to CKD transition.

**Figure 6:**
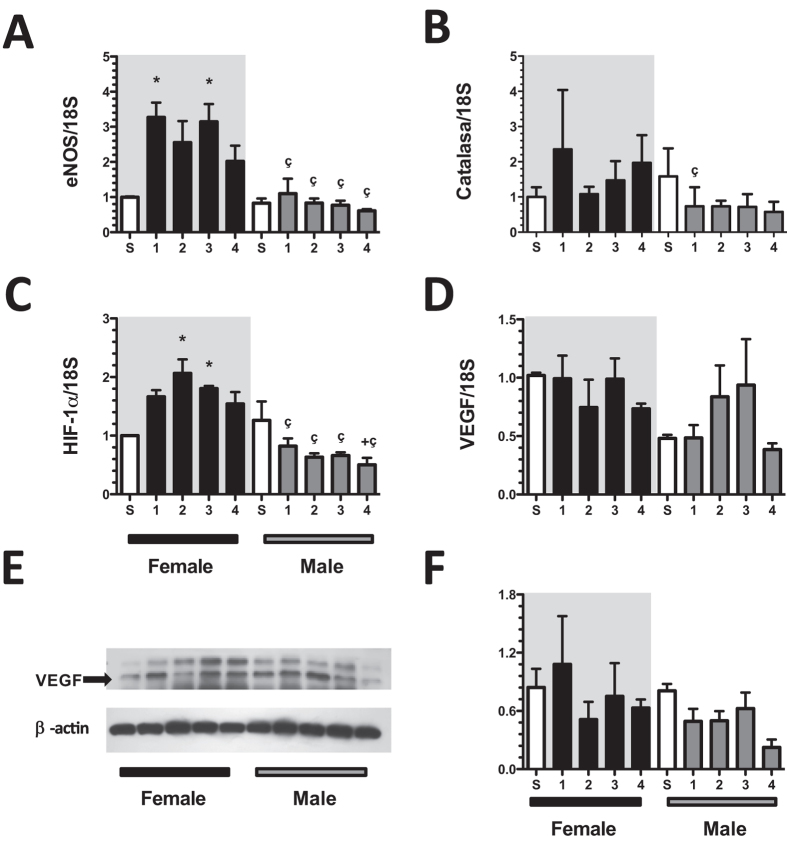
Dimorphic response of some mediators involved in the AKI to CKD transition. (**A**) eNOS mRNA levels, (**B**) catalase mRNA levels, (**C**) HIF1α mRNA levels, (**D**) VEGF mRNA levels, (**E**) Representative autoradioghraphies of VEGF and β-actin Western Blot analysis, and (**F**) VEGF protein levels. Female groups are in a gray background, in which sham is represented by white bars and F + IR group in black bars, following by sham male in white bars and M + IR group in gray bars. The mRNA levels were determined at least by duplicate (n = at least 4 per group). Data are shown as mean ± SE. *p < 0.05 vs, Sham female group, ^+^p < 0.05 vs. sham male group, and ^ç^p < 0.05 vs. F + IR group.

**Figure 7:**
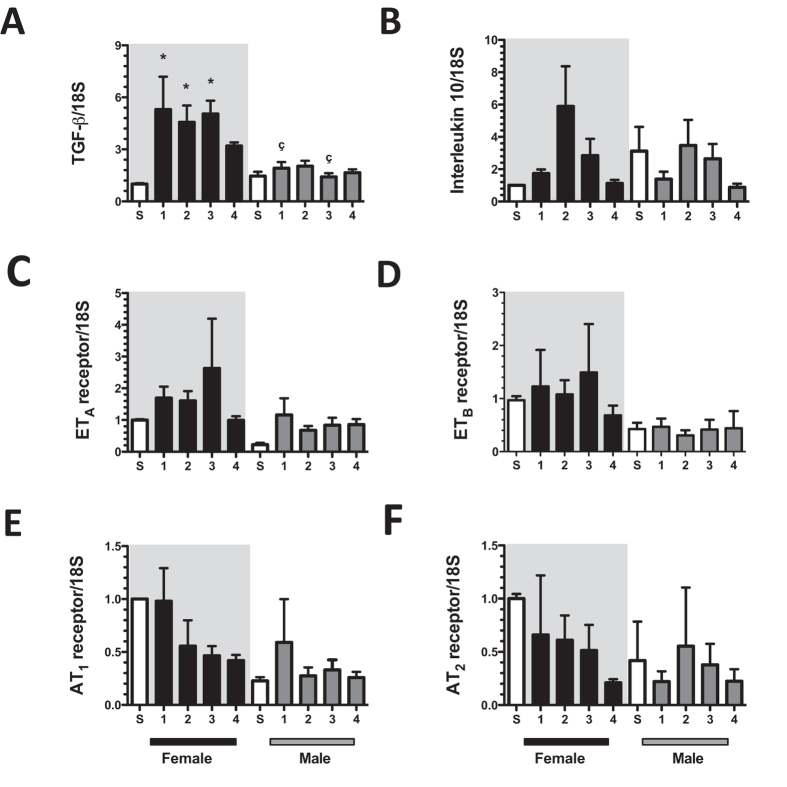
Anti-inflammatory and vasoactive pathways mRNA levels in the dimorphism found in the progression of CKD induced by AKI. (**A**) TGF-β mRNA levels, (**B**) Interleukin 10 mRNA levels, (**C**) ET_A_ receptor mRNA levels, (**D**) ET_B_ receptor mRNA levels, (**E**) AT_1_ receptor mRNA levels, (**F**) AT_2_ receptor mRNA levels. Female groups are in a gray background, in which sham is represented by white bars and F + IR group in black bars, following by sham male in white bars and M + IR group in gray bars. Groups included at least 4 rats per group. Data are shown as mean ± SE. *p < 0.05 vs, Sham female group, ^+^p < 0.05 vs. sham male group, and ^ç^p < 0.05 vs. F + IR group.

In order to know the mechanisms responsible in the renoprotection observed in female rats, we evaluated the mRNA levels of several signal pathways involved in the pathophysiology of CKD. The endothelial nitric oxide synthase (eNOS) mRNA levels were significantly increased in the F + IR group at 1^st^ and 3^rd^-months after IRI compared to sham group. In contrast, mRNA levels of eNOS remained unaltered in the M + IR groups, but were significantly different than those observed in F + IR groups (Fig. 6A). There were not changes in catalase mRNA levels among the groups, except for the 1^st^-month in M + IR, in which a significant reduction was observed compared to F + IR group (Fig. 6B). We also found a significant increase in hypoxia-inducible factor (HIF1α) mRNA levels in the F + IR group at the 2^nd^ and 3^rd^-month post-ischemia, contrasting with the behavior in the M + IR group that was significantly smaller than the corresponding F + IR groups and different from S male group at 4^th^-month (Fig. 6C). Although, the VEGF mRNA levels were similar during the time-course of the study in the F + IR and M + IR groups (Fig. 6D), the VEGF protein levels towards to be lower in M + IR group during the follow-up, but a significant difference was only found at the fourth month (Fig. 6E-F), Finally, the mRNA levels of anti- inflammatory cytokines and vasoactive receptors were evaluated. There was a significant increase in TGF-β mRNA levels in the F + IR group, since the 1^st^-month post-ischemia and this effect was not seen in the M + IR groups (Fig. 7A). Similarly, there was a trend to increase of interleukin-10 mRNA levels in the F + IR groups (Fig. 7B). No differences were found in the mRNA levels of vasoactive factors (Fig. 7C,E and F), except in ET_B_ receptor in which the values tended to be lesser in male groups (Fig. 7D).

## Discussion

In this study, we show that after 24-h that of inducing renal injury by 45 min of renal bilateral ischemia, female and male rats exhibited a similar extent of IRI, but after 4 months of the initial IRI, the M + IR group, but not the F + IR group, exhibited clear signs of CKD, characterized by proteinuria, increased oxidative stress and structural changes such as: glomerular hypertrophy and tubulointerstitial fibrosis. These findings provide evidence to support that the F + IR group were protected from the development of CKD, even though they had a similar degree of initial AKI (Fig. 1). Moreover, when the female rats were oophorectomized, this group exhibited proteinuria and oxidative stress as the M + IR group. These results indicate that sexual dimorphism observed can be attributed to sex hormones, since, the oophorectomy generated a similar behavior of the AKI to CKD transition in female rats to that observed in male rats.

Several studies have shown that renal response to different pathological processes is different between males and females animals and it has also been observed in humans. Specifically, it has been reported that renal disease in males is associated with faster progression independently of differences in blood pressure^29,33–42^. When it comes from ischemia/reperfusion the observations are not consistent. Studies conducted in Wistar rats have found that functional and structural injuries induced by ischemia/reperfusion are worsen in the male than female^43–46^, similar between female and male^47,48^, or even worsen in female than male rats^49,50^, and these results differ to that occurs in mice in which the females are more resistant to IR injury^39^. Using renal function (creatinine clearance and renal blood flow), and tubular injury markers (proteinuria, injured tubules %, and urinary excretion of Hsp72), we found, that after 24-h of renal bilateral ischemia, female and male rats displayed the same magnitude of IRI. Therefore, sexual dimorphism in IRI after 24-h was not observed, except in the oxidative stress, because the F + IR did not exhibit elevation of UH_2_O_2_V, as was observed in the M + IR group (Fig. 1D). This finding on oxidative stress was further explored in the renal tissue by measuring renal GSH content. The M + IR indeed exhibited a significant reduction in renal GSH, an effect that was not observed in the F + IR group (Fig. 2A), despite the extensive tubular proximal injury (Fig. 1). These results indicate that female hormones seems to maintain the GSH content in extreme conditions and they also are able to protect for their powerful antioxidant activity, as has been previously demonstrated^51^. Although female exhibited a lesser oxidative stress than male rats, it was not enough to reduce IR injury, because several players take place into AKI pathophysiology, mainly: 1) enhanced vasoconstriction, due to an imbalance in the release of vasoconstrictor and vasodilator factors; 2) endothelial cell injury, that promotes activation and transmigration of leucocytes which are able to produce cytokines and a pro-inflammatory state; 3) and epithelial cell injury by also contributing to the inflammation through releasing chemotactic cytokines, and by the loss of the tubular integrity that provokes a reduction in the survival pathways^8^.

The CKD progression after IRI was observed in the M + IR group accordingly with our previous studies^23,26,32^, but interestingly, female rats did not develop CKD.

Among the potential mechanisms regulated by sex hormones are: changes in renal hemodynamics, and altered vasoactive factors release, transcription factors, pro-fibrotic and pro-inflammatory cytokines^52^. Moreover, female hormones may influence the defense in response to pathophysiological events by its antioxidant property^49,53^. The antioxidant effect of estrogen is mediated by the hydroxyl group at the C3 position of the A ring of the steroid molecule and has been reported that ouabain, an inhibitor of Na^+^/K^+^ ATPase blocks these protective effects, suggesting that the antioxidant effect helps to maintain the function of this pump reducing the accumulation of intracellular sodium^54^. In fact, our study of the time-course of UH_2_O_2_V in the AKI to CKD transition, showed that oxidative stress was higher in the M + IR than in the F + IR group, an effect that was seen in a very early phase post-ischemia and remained along the study (Figs 1D, 2A, and 3C). The lower oxidative stress in female rats exposed to IRI, was not seen when the rats were oophorectomized (Fig. 5F). However, a limitation of the present study is that it cannot exclude that the present observations are characteristic of Wistar rats only. A broader study would be beneficial.

Endothelial dysfunction is caused by reduced levels of nitric oxide (NO) derived from the endothelium. It has been reported that renal diseases are associated with reduced NO synthesis provoked by the reduced eNOS expression or activity^55^. We found that during the AKI to CKD transition, the M + IR group have similar eNOS mRNA levels throughout the study. In contrast, them were significantly increased in the F + IR group very early after IRI. These results indicate that female rats besides having better antioxidant response, also may generate more NO, which was associated with the disease prevention.

Under conditions of low oxygen tension, HIF1α plays an essential role in regulating several of its target genes to mediate actions on: cell proliferation, angiogenesis, apoptosis, etc.^56^. HIF1α regulates angiogenesis by increasing the expression of VEGF. During the AKI to CKD transition there is a chronic hypoxia and a reduction in the peritubular capillaries^19,57^. These findings suggest that HIF1α signaling may be affected during this transition. In fact, we observed that the greatest damage in the M + IR group was associated with a significant reduction in HIF1α and VEGF protein levels. Whereas a dimorphic response was observed in the F + IR group, because HIF1α mRNA levels was enhanced after IRI since the 1^st^-month, reaching statistical difference by ANOVA after the 2^nd^-month. These results suggest that another mechanism by which female rats did not progress to CKD is mediated by its ability to increase HIF1α since early stages after IRI, which in turn could help to prevent vascular rarefaction, chronic hypoxia and renal fibrosis.

In addition to this, it has been observed that estrogens have anti-fibrotic and anti-apoptotic properties in the cardiomyocytes^58^. Also, it has been reported that administration of 17-beta estradiol to hypertensive oophorectomized rats attenuates glomerulosclerosis and tubulointerstitial fibrosis^59^ while, in rats with type II diabetes, this hormone protects podocytes by increasing estrogen receptor beta^35^. This effect on podocytes, apparently is mediated by stabilizing the cytoskeleton of these cells^60^. On contrary, it has been postulated that testosterone has fibrotic and apoptotic properties through increasing TNFα signaling^37^. Accordingly, with this evidence, the M + IR group developed glomerular hypertrophy and tubule-interstitial fibrosis after IRI and the female rats do not. Surprisingly, the time-course of TGF-β mRNA levels after the ischemic insult showed a clearly dimorphic response. In the F + IR, TGF-β mRNA levels were significantly increased, since the 1^st^- month and remained elevated along the study, whereas in the M + IR group this anti-inflammatory response did not occur. Similarly, interleukin-10 only trend to increase in the F + IR groups. Although several studies have revealed the fundamental role of TGF-β in renal fibrosis^61^, has also been observed that the use of antibodies against TGF-β in models of diabetic nephropathy^62^, or in puromycin aminonucleoside induced nephropathy^63^ worsen proteinuria. In fact, Wang W *et al*. showed that TGF-β can relieve the inflammation through Smad7^64^. Given the significant tubulointerstitial fibrosis in male rats compared with females, we believe that the early elevation of this cytokine in female rats, could be exerting a renoprotector effect. In support of this, Klempt *et al*. reported that TGF-β is induced after cerebral ischemia and this rise was associated with post-axial repair^65^. Furthermore, it has been reported that TGF-β anti-inflammatory properties are mediated by promoting polarization of anti-inflammatory Th2 macrophages in cerebral malaria^66^. Therefore, the increment in TGF-β mRNA levels in early stages after ischemia observed in the F + IR group is another beneficial mechanism that females install after the IRI.

In summary, there is a sexual dimorphism in the AKI to CKD transition and the renoprotection observed in the F + IR group was lost with the oophorectomy: These results strongly suggest that female sex hormones are responsible of the renoprotection observed. Within the renoprotective mechanisms that installed females after IRI are: best antioxidant and anti-inflammatory defense, as well as higher HIF1α and eNOS mRNA levels.

## Methods

All experiments involving animals were conducted in accordance with NIH Guide for the Care and Use of Laboratory Animals and with the Mexican Federal Regulation for animal reproduction, care, and experimentation (NOM-062-ZOO-2001). The study was approved by the Animal Care and Use Committees: Comité para el cuidado y uso de animales de laboratorio, Instituto de Investigaciones Biomédicas and for Comité de investigación en animales, Instituto Nacional de Ciencias Médicas y Nutrición.

### Experimental Protocol 1

Thirty-nine female (250–280 g) and thirty-nine (290–310 g) male Wistar rats were included and divided into seven groups: Two groups of control animals underwent sham surgery that were studied and sacrificed at 24-h (5 females and 5 males) and at 4-months (4 females and 4 males); and 5-groups who underwent bilateral renal ischemia for 45 min and were studied at 24-h (6 females and 6 males) and at 1 (5 females and 5 males), 2 (6 females and 6 males), 3 (5 females and 5 males), or 4-months post-ischemia (8 females and 8 males), F + IR and M + IR, respectively. The bilateral renal ischemia in the female rats was performed during the estrogenic phase. This phase was evaluated by vaginal smear, briefly cyclicity was recorded following examination of vaginal smears for the proportions of leucocytes, epithelial cells and cornified cells in the smear. The smears were assessed and cycles identified as described previously^67^.

### Experimental Protocol 2

Fifty-five female Wistar rats were oophorectomized at 70 days of age and allowed to evolve 1-month until estrogen levels decreased and were divided into 7-groups: two groups of control animals underwent sham surgery that were studied and sacrificed at 24-h (n = 8) and at 4-months (n = 9); and five groups who underwent bilateral renal ischemia for 45 min and were studied at 24-h (n = 8), and at 1 (n = 8), 2 (n = 6), 3 (n = 8), or 4-months post-ischemia (n = 8).

### Ischemia/reperfusion model

Rats were anesthetized with an intra-peritoneal injection of sodium pentobarbital (30 mg/kg) and placed on a heating pad to maintain core body temperature at 37 °C. Renal pedicles were isolated, and bilateral renal ischemia was induced using a non-traumatic clamp on each renal artery for 45 min. Reperfusion was achieved by release of the clips and confirmed by return of oxygenated blood to the kidney. The muscle and the skin were closed with 3-0 vicryl and silk sutures, respectively. For sham surgery, laparotomy and renal pedicle dissection, without clamping, was performed in anesthetized rats.

### Bilateral Oophorectomy

The animals were anesthetized by intraperitoneal injection of sodium pentobarbital at a dose of 30 mg/Kg and were placed in a heating bed. Double dorsolateral incision was performed and the oviducts were ligated and removed; then, the abdominal wall and skin were sutured. After surgery, the animals remained in the heating bed until their anesthesia recovery. One month after, rats underwent to bilateral renal ischemia (45 min) and studied either, 24-h or 4-months after.

### Functional Studies

At the end of the experimental period, rats were anesthetized with sodium pentobarbital (30 mg/kg) and placed on a homoeothermic table. The femoral arteries were catheterized with polyethylene tubing (PE-50). The mean arterial pressure (MAP) was monitored with a pressure transducer (model p23 db, Gould) and recorded on a polygraph (Grass Instruments, Quincy, MA). An ultrasound transit-time flow probe (transonic flowprobe, New York, NY) was placed around the left artery and filled with ultrasonic coupling gel (HR Lubricating Jelly, Carter-Wallace, New York, NY) to record the renal blood flow (Transonic flowmeter, New York, NY). Blood samples were taken at the end of the study.

### Biochemical Studies

Proteinuria was determined monthly from 24-h urine collections using the turbidimetric method with trichloroacetic acid (TCA) throughout the monthly follow-up in all studied groups.

Urine and serum creatinine concentrations were measured with Quantichrom creatinine assay kit (DICT-500), and renal creatinine clearance was calculated.

Serum estradiol concentrations were determined by radioimmune analysis using a commercial rat kit, (DPC Coat-a-count, TKE21, Diagnostic Products, CA, USA).

### Light microscopy analysis

For the light microscopy, the left kidney was perfused through the femoral catheter with PBS and then 4% neutral buffered formalin, and perfusion was continued until fixation was completed, maintaining the MAP that each rat had during the experiment. Renal tissue was paraffin embedded, 3 µm sections were stained with periodic acid Schiff (PAS) or Sirius red. For the acute study, ten subcortical fields (magnification ×100) were recorded from each kidney slide using a digital camera incorporated in a Nikon Light microscope. The injured tubules were analyzed blindly. Tubular damage was characterized by a loss of brush border, lumen dilation, and detachment from basement membrane. In each microphotograph, injured tubules were counted, and the results were expressed as the average of fields observed. For the chronic study, the glomerular diameter and area was measured in at least 50 glomeruli per rat, as we previously reported^23^. For this purpose, ten to fifteen images of different renal cortex fields were recorded (magnification ×100). In another ten images (magnification ×400), tubular width was measured in at least 100 tubules. Tubulointerstitial fibrosis consisted in extra cellular matrix expansion with collagen deposition together with distortion and collapse of the tubules; fibrosis was evidenced by red coloration in Sirus red stained slides. The degree of tubulo-interstitial fibrosis was measured by morphometry in five to eight subcortical fields (magnification ×400). The affected area was delimited and the percentage of tubulo-interstitial fibrosis was calculated by dividing the fibrotic area by the total field area, excluding the glomerular area. All the analyses were performed blinded.

### Urinary hydrogen peroxide assay

The amount of hydrogen peroxide (H_2_O_2_) in urine was determined with an Amplex Red Hydrogen Peroxide/Peroxidase Assay Kit (Invitrogen, Eugene, OR) according to the manufacturer’s instructions.

### Glutathione (GSH) and glutathione disulfide (GSSG) levels

#### Tissue Preparation

The right kidney was excised and separated into renal cortex and medulla. The sections were maintained in ice-cold saline solution (0.9% NaCl). A 10% whole homogenate was prepared in ice-cold homogenization buffer [154 mM KCl, 5 mM diethylenetriaminepentaacetic acid (DTPA), and 0.1 M potassium phosphate (KPi) buffer, pH 6.8]. Immediately after, one volume of cold acid buffer consisting of 40 mM HCl, 10 mM DTPA, 20 mM ascorbic acid, and 10% trichloroacetic acid (TCA) was added to one volume of homogenate. The suspension was centrifuged at 14,000 rpm and the resulting supernatant solution was maintained at −70 °C for at least 4 weeks.

#### O-phthalaldehyde assay (OPA) Assay Procedure

The following solutions were required to perform the OPA assay: redox quenching buffer (RQB) (20 mM HCl, 5 mM DTPA, 10 mM ascorbic acid); 5% TCA in RQB (TCA–RQB); 7.5 mM N-ethylmaleimide (NEM) in RQB; 1.0 M KPi buffer (pH 7.0); 0.1 M KPi buffer (pH 6.9); 100 mM dithionite (DT; sodium hydrosulfite) in RQB; 5.0 mg/ml OPA in methanol. DT and OPA solutions were prepared immediately before use. Standards were prepared as follows: 0.1 mM GSSG in TCA–RQB; 0.1 mM GSH in TCA–RQB. The trace levels of GSSG were removed by treating 1.0 ml of a 1.0 mM solution of GSH in TCA–RQB with 25 mg of zinc dust. The assays consisted of paired samples labeled A and B. Sample A was the background consisting of non-glutathione-dependent fluorescence that was subtracted from the paired sample B. From GSSG 1 mM stock, 20 μl was taken and 980 μl of TCA-RQB was added to make the standard curve. OPA-derived fluorescence was measured at 365-nm excitation (slit width 5 nm) and 430-nm emission (slit width 20 nm)^68^.

### eNOS, catalase, HIF1α, VEGF, ETA, ETB, AT1, AT2, TGF-β, and IL-10 mRNA levels

The right kidney was removed and quickly frozen. The total renal cortex RNA was isolated from the kidneys using the TRIzol method (Invitrogen, Carlsbad, CA) and checked for integrity using 1% agarose gel electrophoresis. To avoid DNA contamination, total RNA samples were treated with DNAase (DNAase I; Invitrogen). Reverse transcription (RT) was carried out with 1 μg of total RNA and 200 U of Moloney murine leukemia virus reverse transcriptase (Invitrogen). The mRNA levels eNOS, catalase, HIF1α, VEGF, ET_A_, ET_B_, AT_1_, AT_2_, TGF- β and IL-10 were quantified by real-time PCR on an ABI Prism 7300 Sequence Detection System (TaqMan, ABI, Foster City, CA). Primers and probes were ordered as follows: eNOS (Rn02132634_s1), catalase (Rn00560930_m1), HIF1α, (Rn0057756_m1), VEGF (Rn01511602_m1), ET_A_ (Rn00561137_m1), ET_B_ (Rn00569139_m1), AT_1_ (Rn00561409_s1), AT_2_ (Rn00560677_s1), TGF-β (Rn00572010_m1), and IL-10 (Rn99999012_m1). As an endogenous control, eukaryotic 18S rRNA (predesigned assay reagent Applied by ABI, external run, Rn03928990_g1) was used. The relative quantification of each gene expression was performed with the comparative threshold cycle (Ct) method.

### Renal VEGF protein levels

VEGF protein levels were detected by Western blot, using 30 μg of protein in 8.5% SDS-PAGE electrophoresis gel and electroblotted. The membranes were incubated with mouse anti-VEGF antibody (1:1000, ThermoScientific) overnight. Next, the membranes were incubated with a secondary antibody and HRP-conjugated anti-mouse IgG (1:5000, Santa Cruz Biotechnology). The proteins were detected with an enhanced chemiluminescence kit (Millipore) and by radiography. All Western blot analyses were performed within the linear range of protein loads and antibody use. The bands were scanned for densitometric analysis using the UVP EC3 Imaging System and the UVP VisionWorks LS Image acquisition and Analysis Software.

### Urinary Hsp72 levels

Urinary Hsp72 levels were detected by Western blot, each urine was diluted 1:10 in 0.9% saline solution, and 10 μL of each dilution was loaded and resolved by 8.5% SDS-PAGE electrophoresis and electroblotted. The membranes were incubated with mouse anti-Hsp72 antibody (ENZO Life Sciences, 1:5000 dilution) for 2-h and then incubated with a secondary antibody, HRP-conjugated goat anti-mouse IgG (1:5000, Santa Cruz Biotechnology). The proteins were detected using a commercial chemiluminiscence kit (Millipore).

### Statistical analysis

The results are presented as the mean ± SE. The differences among the four studied groups at 24-h and 4-months were assessed by ANOVA using the Bonferroni correction for multiple comparisons. All comparisons passed the normality test. The differences in the ranks of glomerular diameters among the groups were evaluated by contingency analysis, and the differences were assessed using the chi-squared test with the Yates correction. Statistical significance was defined when the p value was <0.05.

The data generated during and/or analyzed during the current study are available from the corresponding author on reasonable request.

## Electronic supplementary material

Supplementary information

## References

[1] Kelly KJ (2006). Acute renal failure: much more than a kidney disease. Semin. Nephrol..

[2] Zappitelli M (2008). Epidemiology and diagnosis of acute kidney injury. Semin. Nephrol..

[3] Friedewald JJ, Rabb H (2004). Inflammatory cells in ischemic acute renal failure. Kidney Int..

[4] Lafrance JP, Miller DR (2010). Acute kidney injury associates with increased long-term mortality. J. Am. Soc. Nephrol..

[5] Go AS (2010). The assessment, serial evaluation, and subsequent sequelae of acute kidney injury (ASSESS-AKI) study: design and methods. BMC. Nephrol..

[6] Basile DP (2007). The endothelial cell in ischemic acute kidney injury: implications for acute and chronic function. Kidney Int..

[7] Basile DP (2011). Impaired endothelial proliferation and mesenchymal transition contribute to vascular rarefaction following acute kidney injury. Am. J. Physiol Renal Physiol.

[8] Bonventre JV, Yang L (2011). Cellular pathophysiology of ischemic acute kidney injury. J. Clin. Invest.

[9] Le DM, Legrand M, Payen D, Ince C (2009). The role of the microcirculation in acute kidney injury. Curr. Opin. Crit Care.

[10] Molitoris BA, Dahl R, Geerdes A (1992). Cytoskeleton disruption and apical redistribution of proximal tubule Na(+)-K(+)-ATPase during ischemia. Am. J. Physiol.

[11] Bedford M, Farmer C, Levin A, Ali T, Stevens P (2012). Acute kidney injury and CKD: chicken or egg?. Am. J. Kidney Dis..

[12] Lozano R (2012). Global and regional mortality from 235 causes of death for 20 age groups in 1990 and 2010: a systematic analysis for the Global Burden of Disease Study 2010. Lancet.

[13] Ferenbach DA, Bonventre JV (2015). Mechanisms of maladaptive repair after AKI leading to accelerated kidney ageing and CKD. Nat. Rev. Nephrol..

[14] Chawla LS, Eggers PW, Star RA, Kimmel PL (2014). Acute kidney injury and chronic kidney disease as interconnected syndromes. N. Engl. J. Med..

[15] Block CA, Schoolwerth AC (2007). Acute renal failure: outcomes and risk of chronic kidney disease. Minerva Urol. Nefrol..

[16] Hsu CY (2008). The risk of acute renal failure in patients with chronic kidney disease. Kidney Int..

[17] Venkatachalam, M. A. *et al*. Acute kidney injury: a springboard for progression in chronic kidney disease. *Am. J. Physiol Renal Physiol* (2010). 10.1152/ajprenal.00017.2010 PMC2867413 20200097

[18] Lewington AJ, Cerda J, Mehta RL (2013). Raising awareness of acute kidney injury: a global perspective of a silent killer. Kidney Int..

[19] Basile DP (2004). Rarefaction of peritubular capillaries following ischemic acute renal failure: a potential factor predisposing to progressive nephropathy. Curr. Opin. Nephrol. Hypertens..

[20] Horbelt M (2007). Acute and chronic microvascular alterations in a mouse model of ischemic acute kidney injury. Am. J. Physiol Renal Physiol.

[21] Chen J (2012). EGFR signaling promotes TGFbeta-dependent renal fibrosis. J. Am. Soc. Nephrol..

[22] Xiao X (2015). Angiotensin-(1–7) counteracts angiotensin II-induced dysfunction in cerebral endothelial cells via modulating Nox2/ROS and PI3K/NO pathways. Exp. Cell Res..

[23] Rodriguez-Romo R (2016). AT1 receptor antagonism before ischemia prevents the transition of acute kidney injury to chronic kidney disease. Kidney Int.

[24] Bechtel W (2010). Methylation determines fibroblast activation and fibrogenesis in the kidney. Nat. Med..

[25] Yang, L., Besschetnova, T. Y., Brooks, C. R., Shah, J. V. & Bonventre, J. V. Epithelial cell cycle arrest in G2/M mediates kidney fibrosis after injury. *Nat. Med*. **16**, 535–543, 531p (2010). 10.1038/nm.2144 PMC3928013 20436483

[26] Barrera-Chimal J (2013). Spironolactone prevents chronic kidney disease caused by ischemic acute kidney injury. Kidney Int..

[27] Kwan G (1996). Effects of sex hormones on mesangial cell proliferation and collagen synthesis. Kidney Int..

[28] Evans M (2005). The natural history of chronic renal failure: results from an unselected, population-based, inception cohort in Sweden. Am. J. Kidney Dis..

[29] Eriksen BO, Ingebretsen OC (2006). The progression of chronic kidney disease: a 10-year population-based study of the effects of gender and age. Kidney Int..

[30] Verhave JC (2003). Cardiovascular risk factors are differently associated with urinary albumin excretion in men and women. J. Am. Soc. Nephrol..

[31] Barrera-Chimal J (2011). Hsp72 is an early and sensitive biomarker to detect acute kidney injury. EMBO Mol. Med..

[32] Barrera-Chimal J (2015). Mild ischemic Injury Leads to Long-Term Alterations in the Kidney: Amelioration by Spironolactone Administration. Int. J. Biol. Sci..

[33] Cattran DC (2008). The impact of sex in primary glomerulonephritis. Nephrol Dial Transplant.

[34] Attia DM (2003). Male gender increases sensitivity to renal injury in response to cholesterol loading. Am. J. Physiol Renal Physiol.

[35] Catanuto P (2009). 17 beta-estradiol and tamoxifen upregulate estrogen receptor beta expression and control podocyte signaling pathways in a model of type 2 diabetes. Kidney Int..

[36] Erdely A, Greenfeld Z, Wagner L, Baylis C (2003). Sexual dimorphism in the aging kidney: Effects on injury and nitric oxide system. Kidney Int..

[37] Metcalfe PD (2008). Testosterone exacerbates obstructive renal injury by stimulating TNF-alpha production and increasing proapoptotic and profibrotic signaling. Am. J. Physiol Endocrinol. Metab.

[38] Muller V (2002). Sexual dimorphism in renal ischemia-reperfusion injury in rats: possible role of endothelin. Kidney Int..

[39] Park KM, Kim JI, Ahn Y, Bonventre AJ, Bonventre JV (2004). Testosterone is responsible for enhanced susceptibility of males to ischemic renal injury. J. Biol. Chem..

[40] Pechere-Bertschi A, Burnier M (2007). Gonadal steroids, salt-sensitivity and renal function. Curr Opin Nephrol Hypertens.

[41] Robert R (2011). Gender difference and sex hormone production in rodent renal ischemia reperfusion injury and repair. J. Inflamm. (Lond).

[42] Verhagen AM, Attia DM, Koomans HA, Joles JA (2000). Male gender increases sensitivity to proteinuria induced by mild NOS inhibition in rats: role of sex hormones. Am. J. Physiol Renal Physiol.

[43] Fekete A (2006). Sex differences in heat shock protein 72 expression and localization in rats following renal ischemia-reperfusion injury. Am. J. Physiol Renal Physiol.

[44] Bazzano T, Restel TI, Porfirio LC, Souza AS, Silva IS (2015). Renal biomarkers of male and female Wistar rats (Rattus norvegicus) undergoing renal ischemia and reperfusion. Acta Cir Bras.

[45] Fekete A (2004). Sex differences in the alterations of Na(+), K(+)-ATPase following ischaemia-reperfusion injury in the rat kidney. J Physiol.

[46] Moslemi F (2016). Effect of angiotensin II type 1 receptor blockade on kidney ischemia/reperfusion; a gender-related difference. J Renal Inj Prev.

[47] Takayama J (2008). Actinonin, a meprin inhibitor, protects ischemic acute kidney injury in male but not in female rats. Eur J Pharmacol.

[48] Afyouni NE (2015). Preventive Role of Endothelin Antagonist on Kidney Ischemia: Reperfusion Injury in Male and Female Rats. Int J Prev Med.

[49] Iran-Nejad A, Nematbakhsh M, Eshraghi-Jazi F, Talebi A (2015). Preventive role of estradiol on kidney injury induced by renal ischemia-reperfusion in male and female rats. Int J. Prev. Med.

[50] Aryamanesh S, Ebrahimi SM, Abotaleb N, Nobakht M, Rahimi-Moghaddam P (2012). Role of endogenous vitamin E in renal ischemic preconditioning process: differences between male and female rats. Iran Biomed J.

[51] Giergiel M, Lopucki M, Stachowicz N, Kankofer M (2012). The influence of age and gender on antioxidant enzyme activities in humans and laboratory animals. Aging Clin Exp Res.

[52] Silbiger SR, Neugarten J (1995). The impact of gender on the progression of chronic renal disease. Am. J. Kidney Dis..

[53] Singh AP, Singh N, Singh Bedi PM (2016). Estrogen attenuates renal IRI through PPAR-gamma agonism in rats. J Surg Res.

[54] Kielar ML (2005). Maladaptive role of IL-6 in ischemic acute renal failure. J. Am. Soc. Nephrol..

[55] Ji H (2005). Sex differences in renal injury and nitric oxide production in renal wrap hypertension. Am. J. Physiol Heart Circ. Physiol.

[56] Shoji K, Tanaka T, Nangaku M (2014). Role of hypoxia in progressive chronic kidney disease and implications for therapy. Curr. Opin. Nephrol. Hypertens..

[57] Tanaka S, Tanaka T, Nangaku M (2014). Hypoxia as a key player in the AKI-to-CKD transition. Am J Physiol Renal Physiol.

[58] Elliot SJ (2007). Gender-specific effects of endogenous testosterone: female alpha-estrogen receptor-deficient C57Bl/6J mice develop glomerulosclerosis. Kidney Int..

[59] Maric C, Sandberg K, Hinojosa-Laborde C (2004). Glomerulosclerosis and tubulointerstitial fibrosis are attenuated with 17beta-estradiol in the aging Dahl salt sensitive rat. J. Am. Soc. Nephrol..

[60] Catanuto P (2012). *In vivo* 17beta-estradiol treatment contributes to podocyte actin stabilization in female db/db mice. Endocrinology.

[61] Meng XM, Nikolic-Paterson DJ, Lan HY (2016). TGF-beta: the master regulator of fibrosis. Nat Rev Nephrol.

[62] Ziyadeh FN (2000). Long-term prevention of renal insufficiency, excess matrix gene expression, and glomerular mesangial matrix expansion by treatment with monoclonal antitransforming growth factor-beta antibody in db/db diabetic mice. Proc. Natl. Acad. Sci. USA.

[63] Ma, L.-J. *et al*. Divergent effects of low versus high dose anti-TGF-Î² antibody in puromycin aminonucleoside nephropathy in rats. *Kidney International* **65**(1), 106–115 (2004). 10.1111/j.1523-1755.2004.00381.x 14675041

[64] Wang W (2005). Signaling mechanism of TGF-beta1 in prevention of renal inflammation: role of Smad7. J Am Soc Nephrol.

[65] Klempt ND (1992). Hypoxia-ischemia induces transforming growth factor beta 1 mRNA in the infant rat brain. Brain Res Mol Brain Res.

[66] Hunt NH, Grau GE (2003). Cytokines: accelerators and brakes in the pathogenesis of cerebral malaria. Trends Immunol.

[67] Rosa ESA, Guimaraes MA, Padmanabhan V, Lara HE (2003). Prepubertal administration of estradiol valerate disrupts cyclicity and leads to cystic ovarian morphology during adult life in the rat: role of sympathetic innervation. Endocrinology.

[68] Senft AP, Dalton TP, Shertzer HG (2000). Determining glutathione and glutathione disulfide using the fluorescence probe o-phthalaldehyde. Anal Biochem.

