# Supplementary material for: Gender Differences in the Acute Kidney Injury to Chronic Kidney Disease Transition
Source: Sci Rep. 2017 Sep 25;7:12270. doi: 10.1038/s41598-017-09630-2 (PMC5612964; doi:10.1038/s41598-017-09630-2)
Supplement: Supplementary file 1 — Supplementary information [file 41598_2017_9630_MOESM1_ESM.pdf]

Gender Differences in the Acute Kidney Injury to Chronic Kidney Disease Transition

Ixchel Lima-Posada, Cinthya Portas-Cortés, Rosalba Pérez-Villalva, Francesco Fontana, Roxana Rodríguez-Romo, Rodrigo Prieto, Andrea Sánchez-Navarro, Guadalupe L. Rodríguez-González, Gerardo Gamba, Elena Zambrano and Norma A. Bobadilla

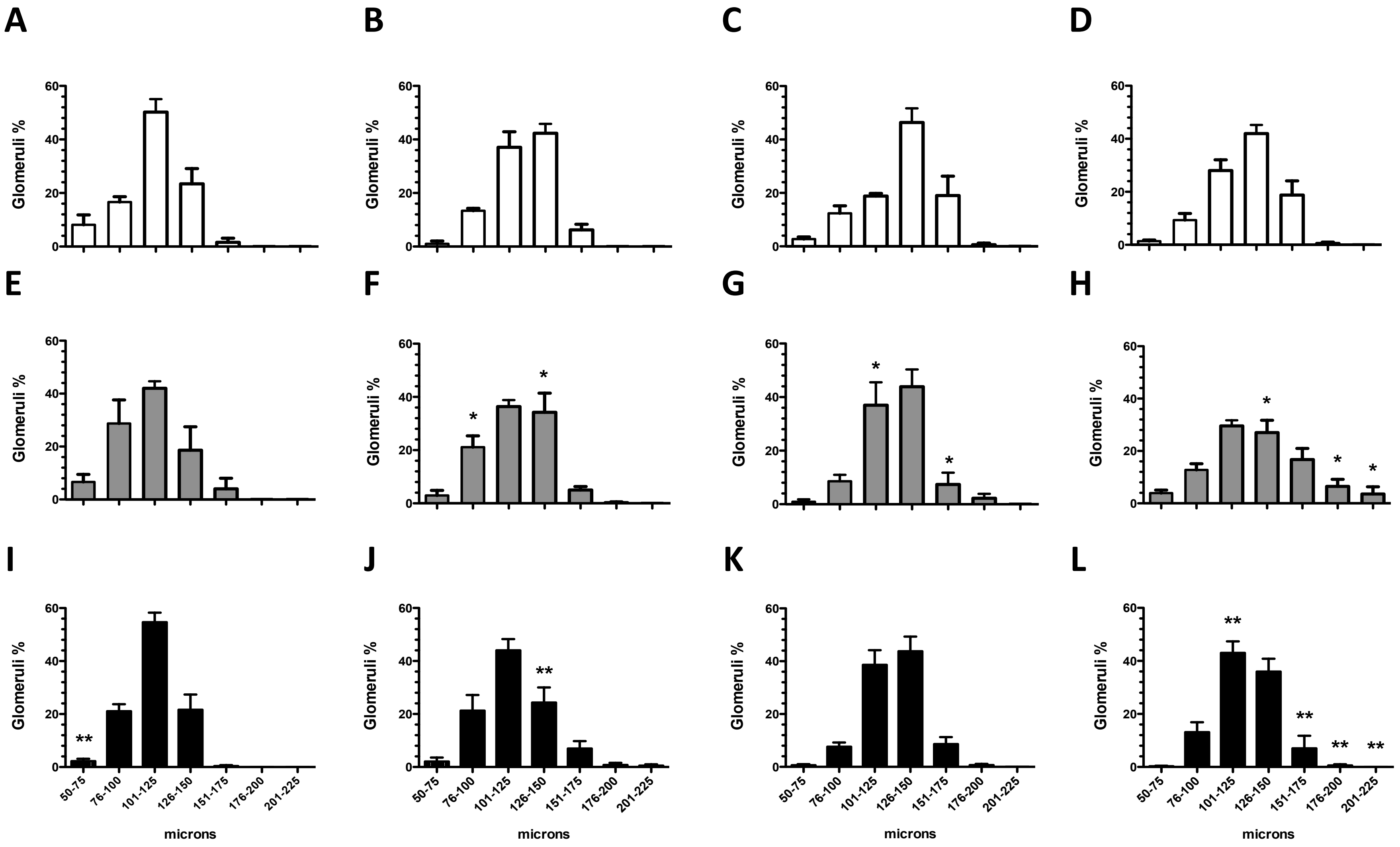

Supplemental Figure 1. Glomerular hypertrophy was seen in males but prevented in females. A-D) Percent of glomeruli in the male control group from 1 to 4-months post-surgery, respectively. E-H) Percent of glomeruli in the M+IR group from 1 to 4-months post-IRI, respectively. I+L) Percent of glomeruli in the F+IR group from 1 to 4-months post-ischemia. Sham male in white bars, and M+IR in gray bars, and F+IR group are in black bars (n=at least 4). The parameters were analyzed by counting at least 100 glomeruli per rat, and data are shown as mean ± SE. *****=p<0.05 vs. male control, and **p<0.05 M+IR group.

Gender Differences in the Acute Kidney Injury to Chronic Kidney Disease Transition

Ixchel Lima-Posada, Cinthya Portas-Cortés, Rosalba Pérez-Villalva, Francesco Fontana, Roxana Rodríguez-Romo, Rodrigo Prieto, Andrea Sánchez-Navarro, Guadalupe L. Rodríguez-González, Gerardo Gamba, Elena Zambrano and Norma A. Bobadilla

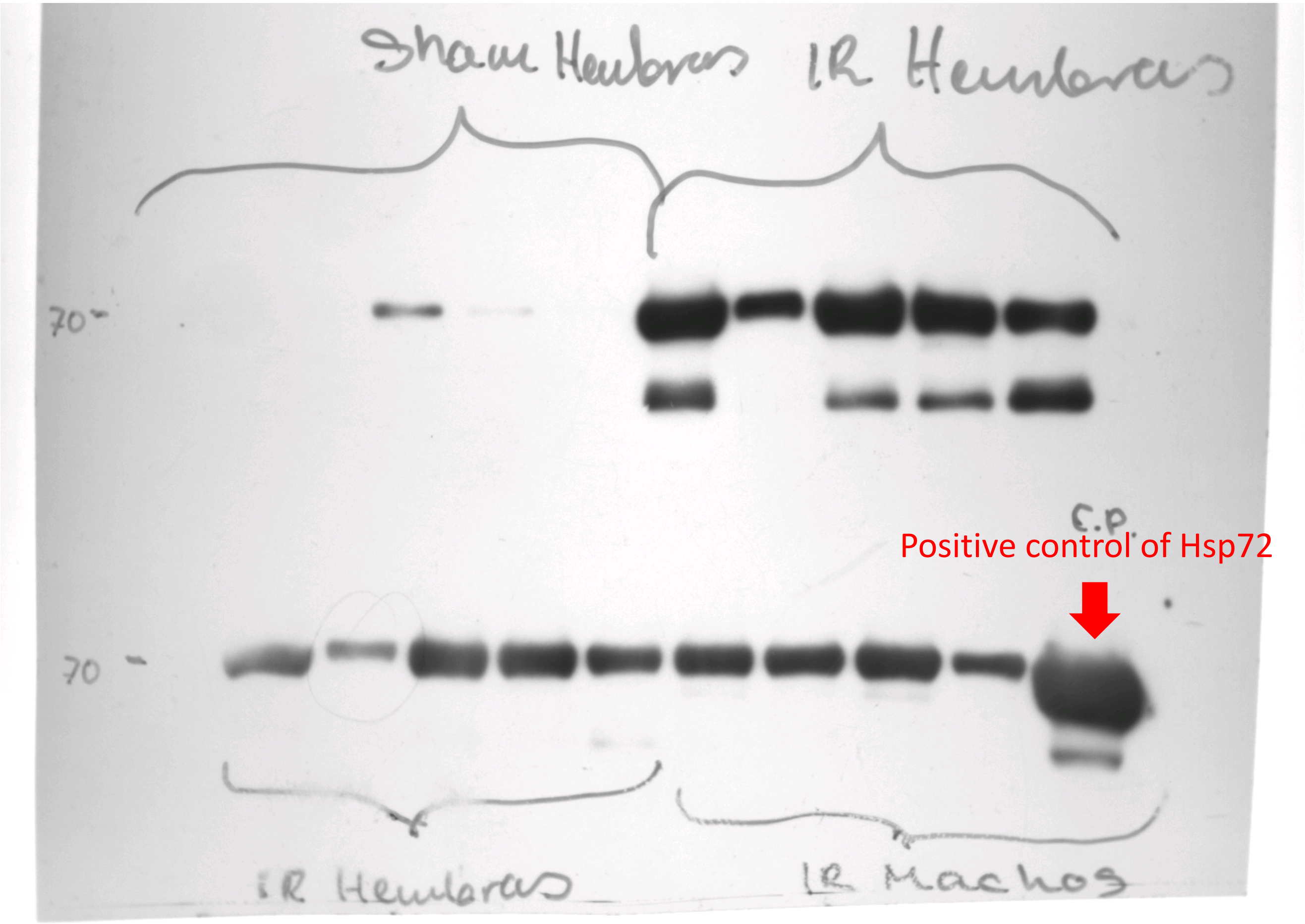

Western Blot Analysis of Hsp72 in the urine of male and females underwent IR that appears in Figure 1.
Exposure time 1 min

Gender Differences in the Acute Kidney Injury to Chronic Kidney Disease Transition

Ixchel Lima-Posada, Cinthya Portas-Cortés, Rosalba Pérez-Villalva, Francesco Fontana, Roxana Rodríguez-Romo, Rodrigo Prieto, Andrea Sánchez-Navarro, Guadalupe L. Rodríguez-González, Gerardo Gamba, Elena Zambrano and Norma A. Bobadilla

VEGF

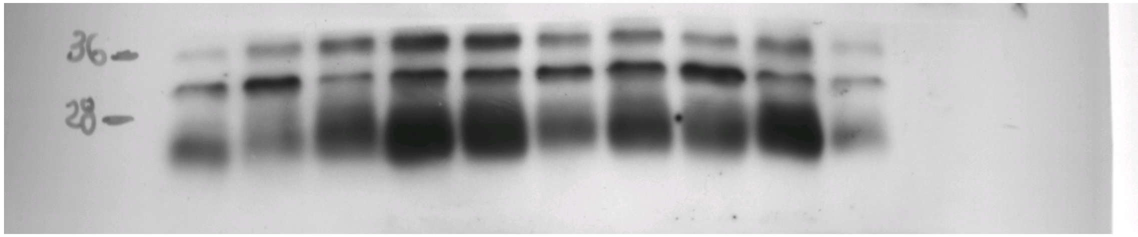

β-actin

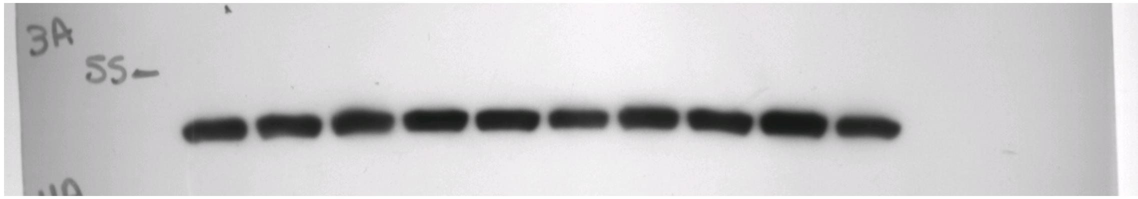

Western Blot Analysis for VEGF and β-actin in the renal cortex, that appears in Figure 6.
Exposure time 30 seconds.
